# EXPLORATORY STRUCTURAL PROCEDURES IN HEALTH SERVICES RESEARCH

**DOI:** 10.1093/geroni/igac059.700

**Published:** 2022-12-20

**Authors:** Martina Roes, Barbara Resnick

## Abstract

When analysing data where little knowledge of relationships is available, exploratory structuring techniques, e.g., clustering and scaling techniques, are applied, using an inductive approach to construct analogies and thus suggest how to organize the data. These procedures in Health Service Research mainly focus on latent variables and are particularly used in the development of instruments or theories. Our symposium includes three presentations on research studies in which exploratory structural procedures were used for data analysis, that were considered appropriate for Health Service Research. Dr. Johannes Bergmann will present a concept mapping technique of the German Preferences for Everyday Living Inventory (PELI-D), for structuring the questionnaire from the perspective of professional nurses. This is a mixed methods participatory approach that combines interpretation and decisions by the researchers with a sequence of multivariate statistical analyses (multidimensional scaling, cluster analysis).. Jan Dreyer will present a Multiple Correspondence Analysis and Hierarchical Cluster Analysis as exploratory data reduction procedures. His aim was to identify types of home-based care arrangements for people living with dementia. To analyse the relationships between care arrangement variables, he performed Multiple Correspondence Analysis. To cluster the care arrangements, he performed Hierarchical Cluster Analysis. Anna Louisa Hoffmann will present an alternative, modified procedure of Multiple Correspondence analysis. Since Multiple Correspondence Analysis underestimates the true quality of data representation, she used Adjusted Multiple Correspondence Analysis to explore construct validity of the Dementia Policy Questionnaire (DemPol-Q). This procedure was considered appropriate to assign categorical variables to latent variables.

